# Image quality assessment in spine surgery: a comparison of intraoperative CBCT and postoperative MDCT

**DOI:** 10.1007/s00701-025-06503-w

**Published:** 2025-03-31

**Authors:** Paulina Cewe, Mikael Skorpil, Alexander Fletcher-Sandersjöö, Victor Gabriel El-Hajj, Per Grane, Michael Fagerlund, Magnus Kaijser, Adrian Elmi-Terander, Erik Edström

**Affiliations:** 1 Department of Trauma and Musculoskeletal Radiology, ME Trauma Radiology, Karolinska University Hospital, 171 64 Stockholm, Sweden; 2 Department of Clinical Neuroscience, Karolinska Institutet, Stockholm, Sweden; 3 Department of Neuroradiology, Karolinska University Hospital, Stockholm, Sweden; 4 Department of Molecular Medicine and Surgery, Karolinska Institutet, Stockholm, Sweden; 5 Department of Neurosurgery, Karolinska University Hospital, Stockholm, Sweden; 6 Institute of Environmental Medicine, Karolinska Institutet, Stockholm, Sweden; 7 Capio Spine Center Stockholm, Löwenströmska Hospital, Stockholm, Sweden; 8 Department of Medical Sciences, Örebro University, Örebro, Sweden; 9 Department of Surgical Sciences, Uppsala University, Uppsala, Sweden

**Keywords:** Cone beam computed tomography, Spine, Neuroradiology, Neurosurgery, Image quality

## Abstract

**Objective:**

To evaluate if intraoperative cone-beam CT (CBCT) provides equivalent image quality to postoperative multidetector CT (MDCT) in spine surgery, potentially eliminating unnecessary imaging and cumulative radiation exposure.

**Methods:**

Twenty-seven patients (16 men, 11 women; median age 39 years) treated with spinal fixation surgery were evaluated using intraoperative CBCT and postoperative MDCT. The images were independently evaluated by four neuroradiologists, utilizing a five-step Likert scale and visual grading characteristics (VGC) analysis. The area under the VGC curve (AUC_VGC_) quantified preferences between modalities. Intra- and inter-observer variability was evaluated using intraclass correlation coefficients (ICC). Image quality was objectively evaluated by contrast and signal-to-noise measurements (CNR, SNR).

**Results:**

In image quality, CBCT was the preferred modality in thoracolumbar spine (AUC_VGC_ = 0.58, *p* < 0.001). Conversely, MDCT was preferred in cervical spine (AUC_VGC_ = 0.38, *p* < 0.004). The agreement was good for inter-observer and moderate in intra-observer (ICC 0.76—0.77 vs 0.60—0.71), *p* < 0.001. SNR and CNR were comparable in thoracolumbar imaging, while MDCT provided superior and more consistent image quality in the cervical spine, *p* < 0.001.

**Conclusion:**

In spine surgery, CBCT provides superior image quality for thoracolumbar imaging, while MDCT performs better for cervical imaging. Intraoperative CBCT could potentially replace postoperative MDCT in thoracolumbar spine procedures, while postoperative MDCT remains essential for cervical spine assessment.

**Key Points:**

- Subjective assessment demonstrated that CBCT was the preferred modality for thoracolumbar spine imaging, while MDCT was favored for cervical spine imaging.
- Agreement between readers was good, while individual readings showed moderate consistency in repeated assessments.
- Objective assessment of image clarity and detail showed both modalities performed equally well in the thoracolumbar spine, while MDCT performed better in the cervical spine.
- Intraoperative CBCT proves superior to postoperative MDCT for thoracolumbar spine imaging, potentially eliminating redundant scans, and improving workflow. Postoperative MDCT remains essential for cervical spine procedures.

**Supplementary Information:**

The online version contains supplementary material available at 10.1007/s00701-025-06503-w.

## Background

In the 1970s intraoperative imaging in spine surgery, including lateral plane radiographs and two-dimensional (2D) fluoroscopy, was first implemented. Cone beam CT (CBCT) was introduced in the 1990s, utilizing multiplanar three-dimensional (3D) technology [46]. While originally developed for dental imaging, CBCT has expanded into various fields including interventional procedures, musculoskeletal radiology, orthopedics and neurosurgery [4, 10, 15, 19, 33]. In spine surgery, intraoperative CBCT has enhanced precision and safety, and has the potential to reduce complications [9]. It has become increasingly integral as it provides imaging data for accurate navigation and allows the surgeon to identify and reposition misplaced screws before finalizing a procedure [3, 7, 50]. Standardized multidetector CT (MDCT) is usually performed postoperatively to confirm and document surgical results [7, 29, 50]. However, imaging utilization has increased significantly, driven by accessibility, reduced costs, and heightened requirements for surgical outcome verification. Balancing diagnostic benefits against cumulative radiation exposure is therefore becoming increasingly important [6, 11, 17, 37]. Postoperative imaging utilizes low-dose (LD) protocols, focused on bone and implant visualization. In such, radiation exposure can be significantly reduced while maintaining diagnostic requirements essential for clinical efficacy [11, 41, 47]. The convergence in image quality between intraoperative and postoperative imaging modalities presents the opportunity to reevaluate traditional imaging workflows and avoid unnecessary repeat examinations. With this comes potential benefits such as reduced cumulative radiation exposure to patients, and more efficient use of healthcare resources [17, 37]. The potential to streamline surgical and diagnostic workflows while enhancing patient safety warrants systematic investigation of the differences and similarities in image quality of intraoperative CBCT and postoperative MDCT in specific surgical applications [3, 7].

We hypothesized that the image and diagnostic quality of CBCT in cervical and thoracolumbar spine stabilization was comparable to MDCT. We evaluated this by subjective and objective assessment of images acquired intraoperatively with CBCT to that of postoperative images acquired with conventional MDCT, in the same patients.

## Methods

### Study characteristics

This retrospective study included patients undergoing spinal fixation for degenerative, deformity, and traumatic conditions. The ethical permit was granted by the local and national ethical authorities (Stockholm: 2018/1490–31 and 2020–00900) that waived the need for consent. The patients included were all above the age of 16 and underwent surgery between December 2016 and July 2018. The thoracolumbar cases were initially and prospectively enrolled in a navigation study which included intraoperative CBCT and postoperative MDCT [14]. The trauma cases were retrospectively selected based on the availability of both intraoperative CBCT and postoperative MDCT imaging data. The CBCT images used in this study were obtained intraoperatively after final placement of implants. The evaluated images included ACDF (with PEEK cages and titanium plates and screws), posterior cervical titanium lateral mass screws and rods, thoracolumbar titanium pedicle screws and rods. All authors had full control of the data, and the information submitted for publication, and none had any conflicts of interest.

### Image acquisition

**Fig. 1:**
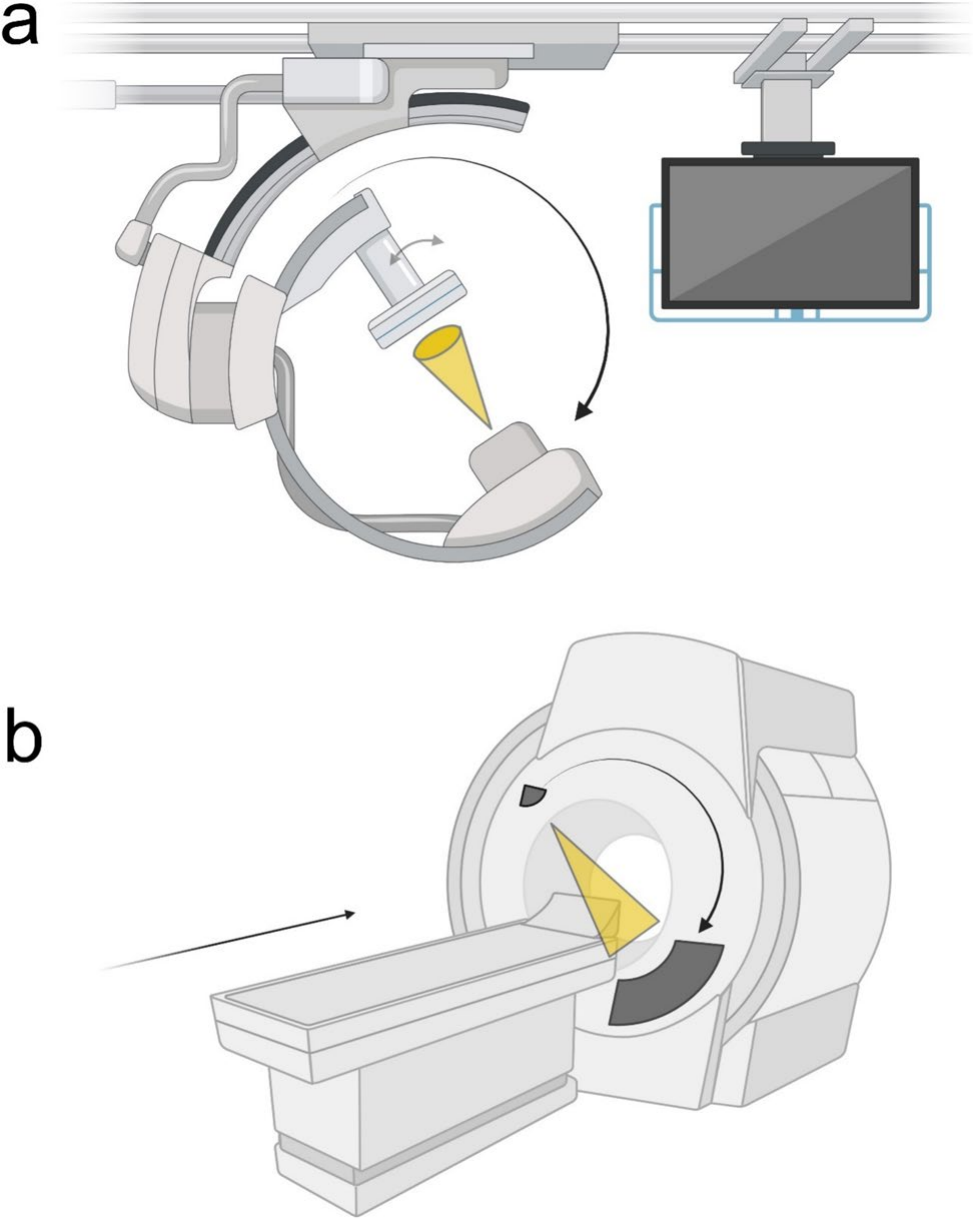
Illustration of the distinct arrangements and functional capabilities of the two imaging technologies in the study. **a** A ceiling-mounted cone-beam computed tomography (CBCT) system used in intraoperative imaging in spinal surgery. During acquisition the C-arm performs a 180° arc around the region of interest and can be tilted ± 20°, indicated by arrows. X-rays originate from the radiation source in a cone shape and the detector captures a volumetric dataset. **b** A multi-detector computed tomography (MDCT) system used in postoperative imaging. During image acquisition, the gantry completes multiple 360° rotations around the region of interest while the table moves accordingly, indicated by arrows. The X-ray beam is fan-shaped, and imaging data is captured on multiple detector rows. Created in BioRender. Cewe, P. (2025) https://BioRender.com/b85h761

For intraoperative 3D CBCT imaging, a ceiling-mounted robotic C-arm (Allura Xper FD20, Philips Healthcare, Best, the Netherlands) was used. CBCT technology utilizes divergent X-rays arranged in a cone shape, originating from the radiation source, with the detector capturing a volumetric dataset during a single rotation around the region of interest (Fig. 1a). The C-arm used in this study performed a 180° arc during acquisition. Depending on the spinal surgery level, three types of routine protocols with different field of view (FOV) were used: small (12.6 × 12.6 cm^2^), medium (17.3 × 17.3 cm^2^), and large (25.2 × 19.5 cm^2^); slice thickness (0.49, 0.45, 0.49 mm), as previously described [12]. Protocols had a fixed tube kilovoltage of 120 kV and a tube current time depending on the automatic dose rate control, which modulates according to the patient’s size. To reduce metal artifacts, the C-arm can be tilted ± 20° to avoid collinearity between the x-ray beam and the direction of the screws.

Postoperative inpatient and outpatient MDCT scans were acquired using either 128- or 256 row MDCT scanners. MDCT technology employs a fan-shaped X-ray beam and multiple detectors within a rotating gantry to capture imaging data (Fig. 1b). Customized protocols that utilized sharp kernel and bone reconstruction algorithms were employed, along with metal artifact reduction (MAR) techniques when available. The mAs were automatically adjusted using automatic tube modulation based on the patient’s body size. Tube voltage was 100–120 kVp for thoracolumbar imaging and 100 kVp for cervical imaging. For both cervical and thoracolumbar imaging, parameters included 0.625 mm slice thickness [7]. CBCT and MDCT scanner parameters are described in Table 1.

**Table 1:** MDCT and CBCT scanner parameters

		Median (IQR)
MDCT	Peak tube voltage (kVp)	120 (100–120)
	Tube current (mA)	92 (54–187)
	Slice thickness (mm)	0.65 (0.63–0.75)
	Rotation speed (s)	0.50 (0.50–0.50)
	Voxel size (mm^3^)	0.71 (0.63–0.75)
	CTDIvol (mGy)	6.6 (1.68–12.51)
CBCT	Fixed tube voltage (kV)	120 (120–120)
	Tube current time (mAs)	*
	Slice thickness (mm)	0.47 (0.45–0.49)
	Voxel size (mm^3^)	0.49 (0.47–0.49)
	Number of CBCT rotations	4 (2–8)
	Effective dose (mSv)	17.8 (4.7–30)

^*^Tube current–time is modulated from 50 to 325 mAs, depending on the patient size

### Image preparation and randomization

**Fig. 2:**
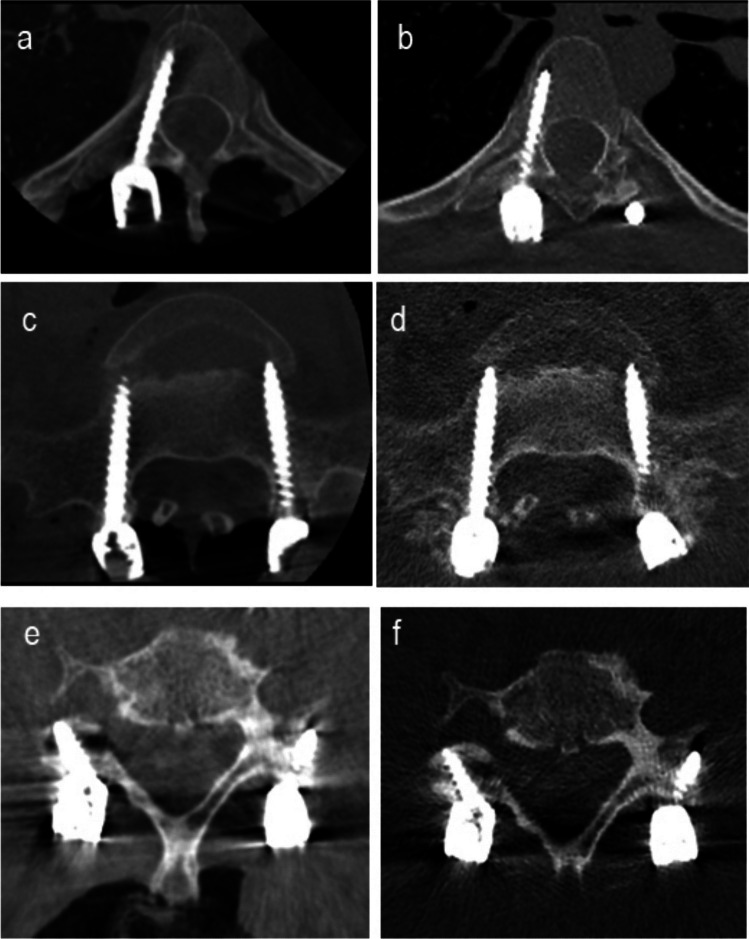
Example of corresponding images of cone-beam computed tomography (CBCT) and multidetector computed tomography (MDCT) images in the axial plane for assessment of image quality in intra- and postoperative spine surgery. Images depict CBCT (**a**,** c**,** e**) and MDCT (**b**,** d**,** f**). Thoracic vertebrae with pedicle screws (**a**,** b**). Lumbar vertebrae with pedicle screws (**c**,** d**). Cervical vertebrae with lateral mass screws (**e**,** f**)

In this study, 207 intraoperative CBCT images were paired with their corresponding 207 postoperative MDCT images; each image set was anonymized, had its DICOM metadata removed, and was cropped to retain only the primary region of interest (Fig. 2). The images underwent four rounds of randomization using Bulk Rename Utility software (TRGNM Software, United Kingdom) and were divided into sets A and B for repeat independent assessment.

### Image assessment protocol

Four neuroradiologists (M.S., M.K., P.G., M.F.) each with more than 10 years of individual experience, conducted independent assessments using Fiji Image J software, in a dark room with a high-resolution screen optimized for diagnostic use to simulate a standard radiologic reading situation [36].

### Reading protocol

Before the study, readers received individual instructions, a sample image set, and a review of the rating criteria for imaging parameters. The scoring followed the European guidelines for CT quality standards [30]. No global harmonization of scores was applied; instead, readers calibrated their individual ratings by evaluating the initial 10 images twice. Repeat readings of all images were conducted after 7–21 days to minimize recall bias and assess intra- and inter-reader agreement. Parameters were evaluated using a 5-point Likert scale ranging from 1 (very poor) to 5 (very good) as outlined in Table 2 [32]. The assessment included the following imaging criteria:

- Image quality
- Cortical bone reproduction
- Trabecular bone reproduction
- Sharpness
- Artifact assessment

**Table 2:** Rating scale for subjective assessment, with descriptive assessment for each imaging parameter

	Image quality	Cortical bone	Trabecular bone	Sharpness	Artifact
Rating scale					
5: Excellent	Diagnostic quality, perfect detail, and easy characterization of anatomic structures and implants	Diagnostic quality, excellent delineation, and detailed structures	Diagnostic quality, excellent delineation, and detailed structures	Diagnostic quality, excellent definition	Diagnostic quality, no artifacts, assignment can be performed
4: Good	Diagnostic quality, anatomic structures, and implants can be well-characterized	Diagnostic quality, good delineation, and structures	Diagnostic quality, good delineation, and structures	Diagnostic quality, good definition	Diagnostic quality, minor artifacts, and assignments can be performed
3: Adequate	Diagnostic quality, anatomic structures, and implants are reasonably well-seen	Diagnostic quality, adequate delineation, and structures	Diagnostic quality, adequate delineation, and structures	Diagnostic quality, adequate definition	Diagnostic quality, more than minor artifacts, assignment can be performed
2: Poor	Detection and characterization of anatomic structures and implants are challenging	Fuzzy/blurry delineation	Fuzzy/blurry delineation	Fuzzy/blurry definition	Moderate artifacts, assignment is hard to perform
1: Very poor	No detection or characterization of anatomic structures or implants is possible	No delineation possible	No delineation possible	No image definition	Completely obstructing visibility

### Objective image assessment

Measurements were obtained from the center of the vertebral body and the paravertebral muscles. A single reader (P.C.) with 5 years’ experience in trauma imaging, performed repeated measurements at every spinal fixation level by placing a 4 mm^2^ region of interest (ROI), at the same location in the vertebral body and paravertebral muscle, on the CBCT image and corresponding MDCT image. Three repeated measurements were taken for each ROI, and mean values were used for calculating SNR and CNR.

Signal-to-noise ratio (SNR) was determined by:

$$SNR = \frac{\left(mean\left(RO{I}_{vertebrae}\right)\right)}{S{D}_{vertebrae}}$$

Contrast-to-noise ratio (CNR) was determined by:

$$CNR = \frac{mean\left(RO{I}_{vertebrae}\right)-mean\left(RO{I}_{muscle}\right)}{\sqrt{S{{D}_{vertebrae}}^{2}+ S{{D}_{muscle}}^{2}}}$$

### Statistical analysis

Observer agreement was assessed using intraclass correlation coefficients (ICC) with 95% confidence intervals. For intra-reader agreement, a two-way mixed-effects model (ICC(3,1)) was used, comparing each reader’s repeated measurements. For inter-reader agreement, a two-way random-effects model (ICC(2,k)) was used to assess agreement among different readers, allowing for generalization to a broader population [27]. Agreement was classified as: poor (< 0.50), moderate (0.50–0.75), good (0.75–0.90), and excellent (> 0.90) according to Koo & Li [25]. Visual grading characteristics (VGC) were analyzed, and the area under the VGC curve (AUC_VGC_) compared modality performance. A curve that was similar or close to being diagonal, resulting in an AUC_VGC_ around 0.5, suggested similarity. An AUC_VGC_ exceeding 0.5 indicated superior performance of the test modality, whereas values below 0.5 suggested superior performance of the reference modality. AUC_VGC_ was calculated for random readers on a trapezoid VGC curve, and binormal curves for plotting, using VGC Analyzer software [20–22]. The program employs bootstrapping to calculate the 95% confidence interval (CI) of the AUC_VGC_ and uses permutation resampling to determine *p*-values for hypothesis testing. Normal distribution was assessed using the Shapiro–Wilk test. Continuous variables with non-normal distributions were expressed as median values accompanied by interquartile ranges (IQR). CNR and SNR were compared using paired t test. Statistical significance was set at *p* < 0.05. Analyses were performed using R (version 4.1.0), R Studio (version 2022.02.0) software, and VGC Analyzer 1.0.3 software.

## Results

### Patient characteristics

In total, 27 patients undergoing cervical- or thoracolumbar spine surgery were included (207 screws; median age 39 (17.0–63.5); 16 men and 11 women). Indications for surgery included congenital, degenerative, and traumatic spine diseases. In the cervical spine, there were 5 flexion-, 2 extension-, 1 Dens-, and 1 Jefferson fracture: median surgical level 4.0 (2.0–4.0). In the thoracolumbar spine, there were 13 scoliosis, 2 spondylolisthesis, 2 kyphosis, and 1 spinal stenosis, median surgical spinal level 8.0 (3.5–11.0). All patients underwent intraoperative CBCT and conventional postoperative MDCT imaging. Median follow-up was 1 (1.00–2.25) days for cervical spine and 9.15 (2.25–12.75) months for thoracolumbar spine surgery (Table 3).

**Table 3:** Patient and surgical characteristics

	Anatomical region	
	Cervical	Thoracolumbar
Patient characteristics		
Women (n)	2	9
Men (n)	7	9
Age (years)	55.0 (52.0–69.0)	18.5 (17.0–43.5)
BMI (kg/m2)	25.0 (21.7–26.7)	20.2 (18.7–21.7)
CBCT-MDCT time (days)	1 (1.00–2.25)	290 (122–413)
Surgery		
Surgical level	4 (2.0–4.0)	8.0 (3.5–11.0)
Flexion fracture (n)	5	
Extension fracture (n)	2	
Dens fracture (n)	1	
Jefferson fracture (n)	1	
Scoliosis (n)		13
Spondylolisthesis (n)		2
Kyphosis (n)		2
Spinal stenosis (n)		1

### Subjective image assessment

**Fig. 3:**
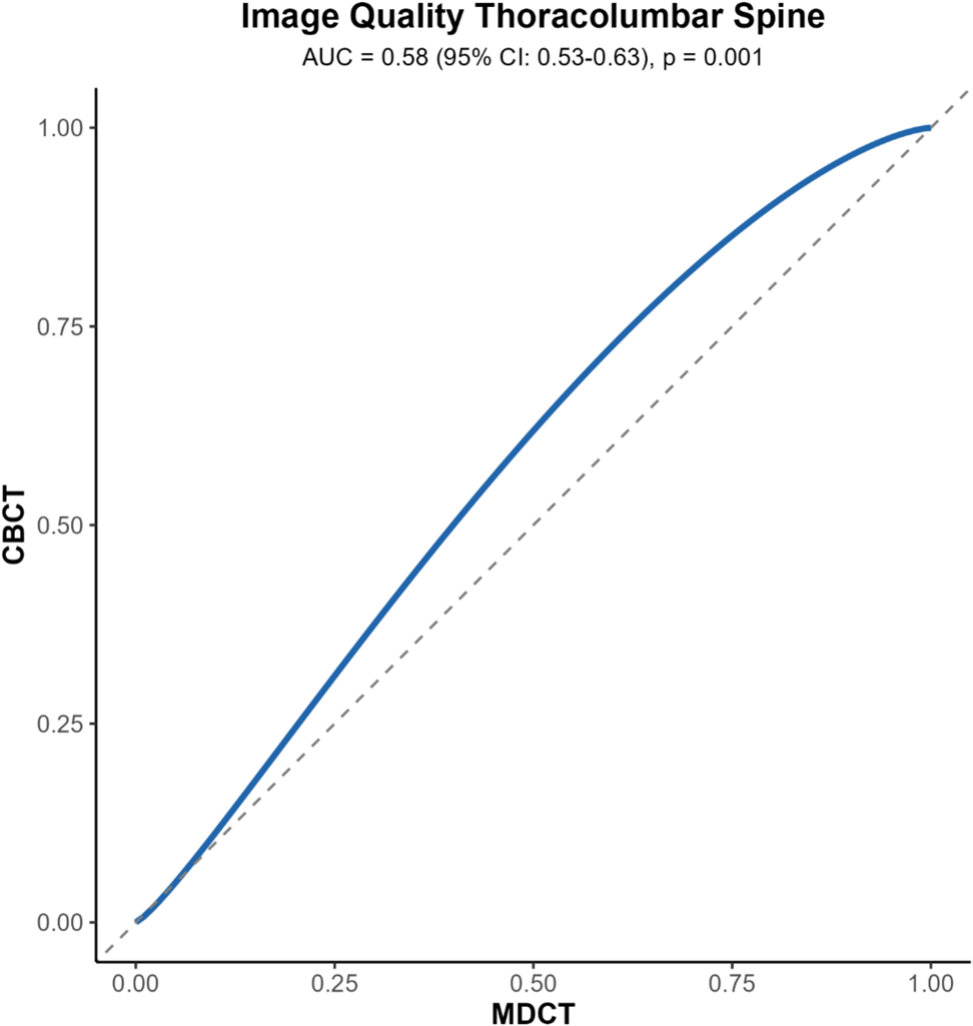
Visual Characteristic (VGC) curve for image quality analysis in the thoracolumbar spine, binormal, random-reader analysis. Greater AUC_VGC_ (> 0.5) indicates better image quality for CBCT compared to MDCT. AUC, area under the curve; CI, confidence interval

AUC_VGC_ indicated that image quality in CBCT in the thoracolumbar spine was superior compared to MDCT (AUC = 0.58, 95% CI, 0.54–0.63; *p* < 0.001). The binormal VGC curve for thoracolumbar spine is presented in Fig. 3. Likewise, sharpness, cortical bone, and trabecular bone were superior in CBCT (AUC = 0.55, 95% CI, 0.50–0.60; *p* < 0.011), (AUC = 0.57, 95% CI, 0.53–0.62; *p* < 0.001), and (AUC = 0.56, 95% CI, 0.51–0.61; *p* < 0.009). A preference for CBCT was observed for artifacts, though this trend was not statistically significant (AUC = 0.53, 95% CI, 0.45–0.59; *p* < 0.251).

**Fig. 4:**
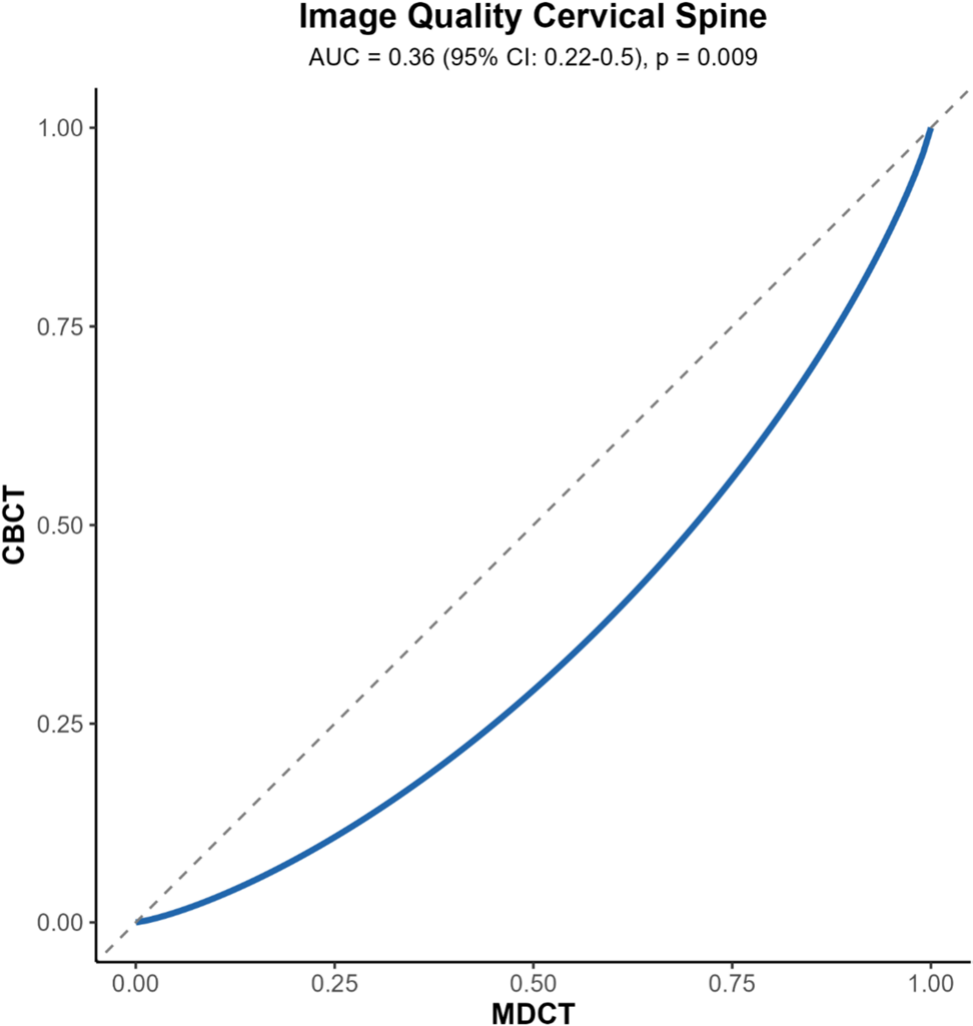
Visual Grading Characteristic (VGC) curve for image quality in the cervical spine, binormal, random-reader analysis. Lesser AUC_VGC_ (< 0.5) indicates better image quality for MDCT compared to CBCT. AUC, area under the curve; CI, confidence interval

In cervical spine imaging, MDCT demonstrated superior image quality compared to CBCT (AUC = 0.38, 95% CI, 0.27–0.50; *p* < 0.004) and in sharpness (AUC = 0.36, 95% CI, 0.25–0.47; *p* < 0.001). The binormal VGC curve for image quality in cervical spine is presented in Fig. 4. Non-significant trends favoring MDCT were observed in the visualization of cortical bone (AUC = 0.46, 95% CI, 0.39–0.53; *p* < 0.185), trabecular bone (AUC = 0.48 95% CI, 0.41–0.56; *p* < 0.669), and artifacts (AUC = 0.43, 95% CI, 0.35–0.51; *p* < 0.051). AUC_VGC_ for all imaging parameters in thoracolumbar and cervical spine are presented in Table 4.

**Table 4:** AUC_VGC_ as a measure of preference in image parameters between CBCT and MDCT in thoracolumbar and cervical spine

	AUC_VGC_	95% CI	*p*-value
Thoracolumbar			
Image Quality	0.58	0.54–0.63	0.001*
Sharpness	0.55	0.50–0.60	0.011*
Cortical	0.57	0.53–0.62	0.001*
Trabecular	0.56	0.51–0.61	0.009*
Artifact	0.53	0.45–0.59	0.251
Cervical			
Image Quality	0.38	0.27–0.50	0.004*
Sharpness	0.36	0.25–0.47	0.001*
Cortical	0.46	0.39–0.53	0.185
Trabecular	0.48	0.41–0.56	0.669
Artifact	0.43	0.35–0.51	0.051

AUC for trapezoid VGC curves, random-reader analysis. AUC_VGC_ indicates preference, values > 0.5 favoring CBCT and values < 0.5 favoring MDCT, for each imaging parameter

*AUC*_*VGC*_ area under the visual grading characteristics curve, *CI* confidence interval

^*^p-value significant

### Intra- and inter-reader variability

**Fig. 5:**
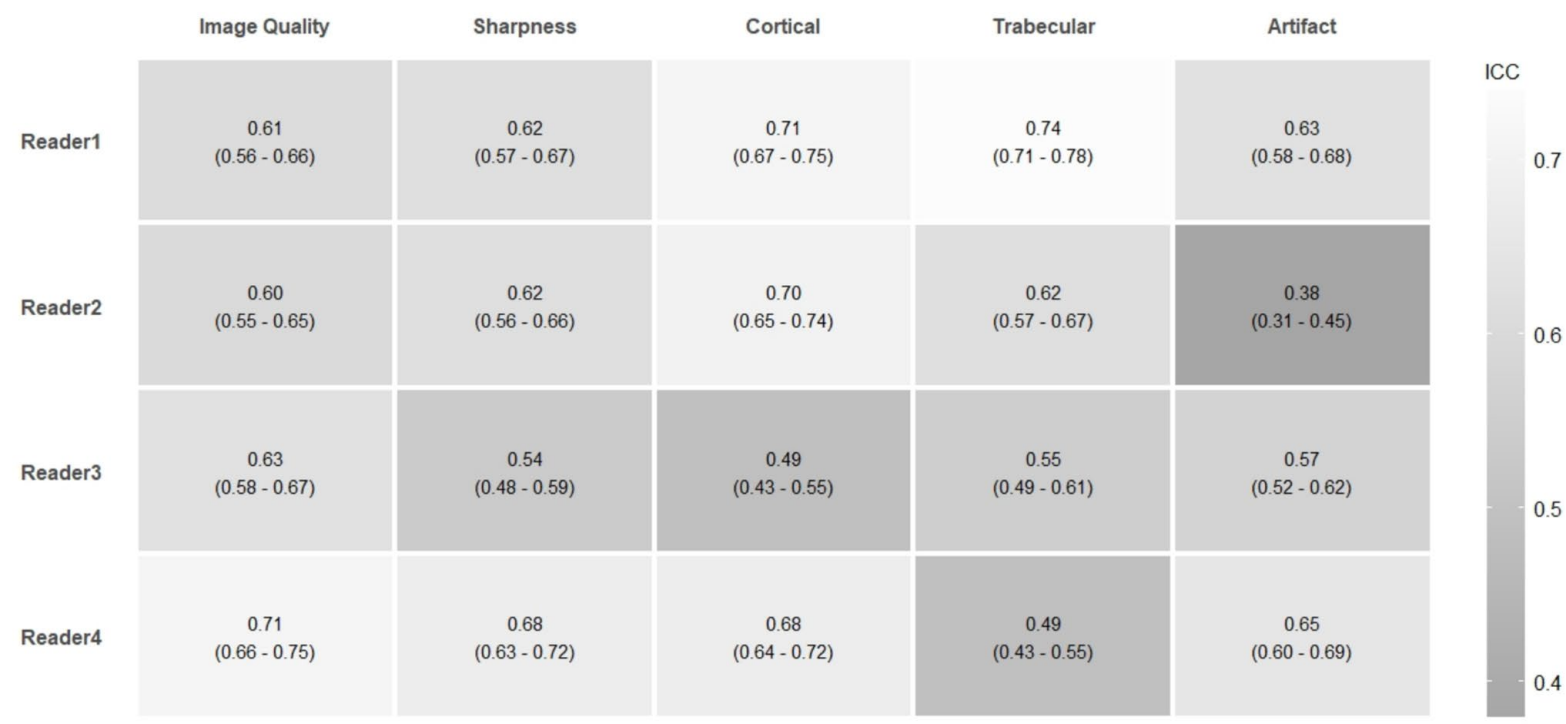
Heatmap representing intra-reader variability for imaging parameter assessments, shown as intraclass correlation coefficients (ICC) with 95% confidence intervals (CI); ICC interpretation: < 0.50 indicates poor reliability, 0.50–0.75 indicates moderate reliability, 0.75–0.90 indicates good reliability, and > 0.90 indicates excellent reliability

Image quality showed good inter-reader (ICC 0.76–0.77) and moderate intra-reader agreement (ICC 0.60–0.71). Similarly, image sharpness showed good inter-reader (ICC 0.76–0.78) and moderate intra-reader (ICC 0.54–0.68) agreement. Cortical and trabecular bone showed moderate inter-reader (ICC 0.67–0.68 and 0.53–0.60) but poor intra-reader agreement (ICC 0.49–0.71 and 0.49–0.74). Similarly, artifact assessment showed moderate inter-reader (ICC 0.56—0.60) but poor intra-reader agreement (ICC 0.38—0.65). All reported values were statistically significant (*p* < 0.001), detailed in Table 5 and Fig. 5.

**Table 5:** Inter-reader variability, data presented as intraclass correlation coefficients (ICC), and 95% confidence intervals (CI)

ICC (95% CI)					
	Image quality	Sharpness	Cortical	Trabecular	Artifact
Session					
1	0.76* (0.67–0.82)	0.78* (0.75–0.81)	0.67* (0.50–0.76)	0.53* (0.29–0.67)	0.60* (0.48–0.69)
2	0.77* (0.72–0.81)	0.76* (0.72–0.79)	0.68* (0.55–0.76)	0.60* (0.38–0.72)	0.56* (0.41–0.66)

Interpretation of ICC: < 0.50 poor reliability, 0.50–0.75 moderate reliability, 0.75–0.90 good reliability, and > 0.90 excellent reliability

*CI* confidence interval, *ICC* intraclass correlation coefficients

^*^p-values < 0.001

### Objective image assessment

In the thoracolumbar spine, the SNR was comparable for both modalities, CBCT was 2.33 (1.99 − 3.69), and MDCT was 2.66 (1.80 − 4.12). Likewise, the CNR values were comparable: CBCT was 1.37 (0.94 − 2.65) and MDCT was 1.41 (1.01 − 2.25).

For the cervical spine, the SNR for CBCT was found to be 0.61 (0.46 − 0.80), which was lower compared to the MDCT value of 2.41 (2.18 − 2.85). The CNR in the cervical region was slightly higher for MDCT of 1.62 (1.50 − 1.84) compared to 1.25 (0.80 − 1.84) for CBCT. All SNR and CNR measurements were statistically significant (*p* < 0.001; Table 6).

**Table 6:** SNR and CNR in cervical and thoracolumbar spine, data in parenthesis represents interquartile range (IQR)

	Cervical spine		Thoracolumbar spine	
	CBCT	MDCT	CBCT	MDCT
SNR (IQR)	0.61* (0.46–0.80)	2.41* (2.18–2.85)	2.38* (1.99–3.69)	2.66* (1.80–4.12)
CNR (IQR)	1.25* (0.80–1.84)	1.62* (1.50–1.84)	1.37* (0.94–2.65)	1.41* (1.01–2.25)

*CNR* contrast-to-noise ratio, *IQR* interquartile range, *SNR* signal-to-noise ratio

^*^p-values < 0.001

## Discussion

This study aimed to determine whether image quality in intraoperative CBCT was comparable to postoperative MDCT in spine surgery. An equivalence between the methods would suggest that the postoperative MDCT could be omitted in many cases.

Four independent neuroradiologists with extensive experience rated both intraoperative CBCT and postoperative MDCT images in cervical and thoracolumbar spine surgery based on five parameters: image quality, cortical bone, trabecular bone, sharpness, and artifacts, using a 5-point Likert scale. VGC analysis assessed imaging parameters, with MDCT as the reference condition. In the thoracolumbar spine, CBCT outperformed MDCT for image quality, sharpness, cortical and trabecular bone. This finding suggests that both modalities perform similarly in diagnostic scenarios, and if no additional information beyond postoperative surgical verification is required, postoperative MDCT could potentially be omitted in thoracolumbar surgery. Conversely, in the cervical spine, MDCT was rated superior in image quality and sharpness compared to CBCT. This discrepancy may partly result from the surgical workflow, where retractors remain in place during the final CBCT acquisition, generating additional metal artifacts that are not present in postoperative imaging. Consequently, these findings underscore the necessity of postoperative MDCT in cervical spine for surgical verification, as intraoperative CBCT alone may not adequately confirm screw placement and fracture realignment.

Analysis of reader agreement revealed differences between the image parameters. Image quality and sharpness showed the highest consistency, with good inter-reader and moderate intra-reader agreement. Artifacts, cortical and trabecular bone showed moderate inter-reader agreement, however poor intra-reader consistency. This disparity between inter- and intra-reader reliability can be attributed to the ICC models used: ICC(2,k) benefits from error averaging across multiple raters, while ICC(3,1) is more susceptible to individual measurement variations. This suggests that multi-reader assessments provide more reliable results than individual readings, indicating the need for enhanced training protocols to improve individual reader consistency and to utilize several readers when performing imaging studies. Furthermore, the lower consistency observed among readers when assessing artifacts, cortical- and trabecular bone underscores the inherent difficulty of subjective image evaluation—particularly in cases involving complex surgical instrumentation and LD protocols—and highlights the need for clearer guidelines and targeted training to improve reader consistency in evaluating these specific parameters.

Both modalities produced similar median SNR and CNR values in the thoracolumbar spine, which indicates that both modalities provided objectively comparable results regarding noise and clarity. In the cervical spine, CBCT had a markedly lower median SNR and slightly lower CNR, indicating more noise and reduced clarity compared to MDCT.

Previous studies have confirmed comparable diagnostic quality of CBCT and MDCT in spine surgery in the thoracolumbar region, when specifically assessing pedicle screw breach and technical assessments of spatial resolution and noise [3, 7, 29, 44]. To our knowledge, no published clinical study has comprehensively compared intraoperative CBCT and postoperative MDCT image quality in spine surgery across the cervical to thoracolumbar regions using both subjective and objective assessments performed by radiologists. Having already studied diagnostic quality in thoracolumbar pedicle screw breach with neurosurgeons, we sought neuroradiologists’ expertise to validate our findings and explore potential workflow improvements [7].

The traditional use of intraoperative 2D fluoroscopy can only detect 52% of misplaced screws compared to CT imaging [35]. Intraoperative 3D imaging, however, allows immediate evaluation and the possibility for correction of misplaced screws, before the patient leaves the OR. Bydon et al., showed that intraoperative 3D imaging enabled revision in 9% of screws corresponding to 35% of the treated patients [8].

As surgical technologies evolve, integrating high-quality imaging modalities during procedures is becoming increasingly important in ensuring optimal surgical outcomes and post-surgical diagnostic verification. MDCT is the standard modality of choice in postoperative imaging in spine surgery [23, 48]. However, recent studies highlight CBCT’s value in specifically musculoskeletal imaging, particularly for fracture detection, as it provides high-quality bone visualization while maintaining low radiation exposure [10, 16, 19, 42]. Minimizing unnecessary imaging is the most effective strategy to reduce radiation exposure. When intraoperative imaging provides comparable quality to postoperative scans, the latter could potentially be eliminated. This can facilitate workflow optimization, offering reduced cumulative radiation exposure, fewer hospital visits, and enhanced resource efficiency [7, 8]. Systematic evaluation of intraoperative CBCT and postoperative MDCT quality, by surgeons and radiologists, is therefore necessary to not only to ensure safety and quality control, but also to avoid unnecessary imaging [12–14].

Image quality depends on several factors, including the technical parameters of the imaging system, patient characteristics, and the specific clinical context in which the imaging is performed [13, 43]. Subsequently, image quality assessment should be task-oriented, multidisciplinary, and clinically relevant rather than solely based on simplified numerical metrics. The clinical assessment protocol should include subjective quality metrics based on radiological image interpretation. The latter involves evaluating images based on various qualitative criteria, such as contrast resolution, anatomical visualization, and artifacts, which can significantly influence diagnostic accuracy and decision-making [43]. To minimize ambiguity in subjective interpretation, it is important to conduct reader performance assessments when comparing modalities. Different methods can be utilized for image evaluation, such as receiver operating characteristic (ROC) analysis, and visual grading characteristics (VGC) analysis, depending on the study performed [5, 21, 22, 40]. Both techniques rely on the reader’s skill and confidence in identifying and interpreting images. The outcomes may vary significantly based on the chosen methodology and the experience level of the readers involved. An understanding of the analysis is important, as it impacts clinical decisions and patient outcomes. ROC analysis focuses on the readers’ ability to discriminate between a test situation and a known ground truth, whereas VGC analysis evaluates subjective, ordinal ratings of a test situation without requiring a strictly binary decision [21, 22, 28]. Consequently, when comparing CBCT and MDCT, VGC analysis serves as a robust alternative to traditional ROC methods by handling ordinal observer ratings, on a predetermined scale, and generating an area under the VGC curve that quantifies differences in perceived image quality and imaging parameters. This method is particularly helpful in multi-reader studies, where individual radiologists may exhibit different scoring patterns. For example, some readers might tend to use the higher and lower end of the scale, while others may cluster their assessments around middle values often referred to as central tendency. By modeling ordinal data directly, VGC analysis reduces the impact of these biases, providing a statistically robust comparison of CBCT and MDCT quality across different readers and scoring styles, and ensuring that genuine differences in image quality are not lost in observer-specific rating tendencies. In our study, VGC analysis was employed to evaluate both overall image quality and additional diagnostic image parameters, providing a more clinically relevant assessment of the imaging techniques used in everyday practice.

Our study’s findings are promising, underscoring the need for additional research. By optimizing intraoperative CBCT imaging protocols, including patient positioning and removing unnecessary surgical material, surgeons may benefit from enhanced image quality, while radiologists could utilize the same images postoperatively for accurate verification of screw placement and assessing fracture realignment. Further investigations should prioritize optimizing intraoperative imaging protocols, identifying appropriate scenarios for omitting postoperative imaging, and developing clear guidelines for when intraoperative imaging alone is sufficient. This evidence-based approach has the potential to enhance clinical efficiency while maintaining high standards of care.

### Limitations and technical considerations

Although assessment of implant placement is an important advantage of intraoperative imaging, this variable was not part of the research question. Nonetheless, in this study, no screws were revised intra- or postoperatively.

Subjective image evaluation is shaped by multiple constraints—such as experience, stress levels, bias, reader fatigue, and the time of day of the reading session—all of which introduce variability into the assessment process [1, 38]. In our study, 414 images were evaluated repeatedly across five imaging parameters using a 5-point Likert scale. Reducing the number of images and parameters might mitigate fatigue and improve assessment consistency. Subsequently, the Likert scale introduces potential tendency bias and limiting the number of score points might have mitigated this issue. Rating harmonization before the study between readers could have been considered. However, thorough calibration and rating harmonization can be time-consuming, and it was deemed preferable to leverage the substantial experience of senior readers over the use of less experienced ones; consequently, independent evaluations by multiple seasoned radiologists were chosen to better reflect real-world clinical interpretation and assess the feasibility of integrating image assessments into routine clinical practice.

Furthermore, patient factors (obesity, poor bone quality) and imaging challenges (low dose protocols, metal screws, surgical instrumentation and patient positioning) can compromise image quality and increase misinterpretation risk [11, 24, 31, 49]. Subsequently, variations in scanner vendors and protocols in outpatient settings, combined with the inherent complexity of these cases, can make consistent evaluation challenging. However, these factors are inherent to clinical practice, making the evaluation in our current study reflective of real-world clinical scenarios.

Finally, while VGC analysis is a robust method for comparing imaging modalities, the intra-reader variability highlights the inherent subjectivity in image quality assessment. Future studies could incorporate automated or semi-automated quantitative metrics to complement subjective evaluations. Building upon this, the evaluation of imaging techniques remains an ongoing challenge, particularly in subjective image analysis. Future improvements in quality assessment may come through standardized scoring systems and advanced tools, including algorithms powered by artificial intelligence (AI) that enhance objectivity and reproducibility [18, 26, 34, 39]. Additionally, dual-energy and photon-counting technologies may further improve image quality by offering superior spatial resolution, material differentiation, and suppressing artifacts [2, 19, 45]. These technical advancements are beyond the scope of the current study, but they hold significant promise for future image quality research and clinical applications.

## Conclusion

In thoracolumbar spine imaging, CBCT outperformed MDCT including image quality and sharpness. Conversely, MDCT proved superior for cervical spine imaging in the same parameters. While both modalities showed comparable SNR and CNR values in thoracolumbar imaging, MDCT demonstrated notably better objective metrics in cervical spine imaging. These findings suggest that intraoperative CBCT could potentially eliminate the need for postoperative MDCT in thoracolumbar procedures, thereby reducing cumulative radiation exposure and optimizing workflows. MDCT continues to be the established standard for postoperative cervical spine imaging.

## Supplementary Information

Below is the link to the electronic supplementary material.

Supplementary file1 (DOCX 256 KB)

## Data Availability

The datasets generated and analyzed during the current study are available from the corresponding author on reasonable request.

## Abbreviations and acronyms

2D: Two-dimensional
3D: Three-dimensional
AI: Artificial Intelligence
AUC: Area Under Curve
CBCT: Cone Beam Computed Tomography
CNR: Contrast to Noise Ratio
FOV: Field of View
LD: Low dose
MAR: Metal Artifact Reduction
MDCT: Multidetector Computed Tomography
OR: Operating Room
ROI: Region of Interest
VGA: Visual Grading Analysis
VGC: Visual Grading Characteristic
SNR: Signal to Noise Ratio

## Declarations

### Study subjects or cohorts overlap

Study subjects or cohorts have been previously reported but not related to this subject, in:

Elmi Terander A, Burström G, Nachabe R et al. (2019) Pedicle Screw Placement Using Augmented Reality Surgical Navigation with Intraoperative 3D Imaging: A First In-Human Prospective Cohort Study. Spine (Phila Pa 1976) 44:517–525.
