# Supplementary material for: Image quality assessment in spine surgery: a comparison of intraoperative CBCT and postoperative MDCT
Source: Acta Neurochir (Wien). 2025 Mar 31;167(1):94. doi: 10.1007/s00701-025-06503-w (PMC11958384; doi:10.1007/s00701-025-06503-w)
Supplement: Supplementary file 1 — Supplementary file1 (DOCX 256 KB) [file 701_2025_6503_MOESM1_ESM.docx]

**Supplemental Table 1** Overall scores of subjective image analysis of CBCT and MDCT, evaluated using a 5-point Likert scales ranging from 1 (very poor) to 5 (very good), data in parenthesis represents interquartile range (IQR).

| **Supplemental Table 1** Overall scores of subjective image analysis of CBCT and MDCT | | | | | | |
| --- | --- | --- | --- | --- | --- | --- |
| **Median (IQR)** | | | | | | |
|  |  | **Image Quality** | **Sharpness** | **Cortical** | **Trabecular** | **Artifact** |
| **Modality** | **Reader** |  |  |  |  |  |
| **CBCT** |  |  |  |  |  |  |
|  | 1 | 2 (2-3) | 3 (2-3) | 2 (2-3) | 3 (3-4) | 2 (2-3) |
|  | 2 | 1 (1-2) | 2 (2-2) | 2 (2-2) | 3 (3-3) | 2 (2-3) |
|  | 3 | 2 (2-2) | 2 (2-3) | 3 (2-3) | 2 (2-3) | 3 (2-3) |
|  | 4 | 2 (2-3) | 3 (2-4) | 3 (2-4) | 3 (3-4) | 3 (2-4) |
| **MDCT** |  |  |  |  |  |  |
|  | 1 | 2 (2-3) | 3 (2-3) | 2 (2-3) | 3 (3-3) | 3 (2-3) |
|  | 2 | 1 (1-2) | 3 (2-3) | 2 (2-3) | 3 (3-4) | 2 (2-3) |
|  | 3 | 2 (2-2) | 2 (2-3) | 2 (2-3) | 2 (2-3) | 2 (2-3) |
|  | 4 | 2 (1-3) | 3 (2-3) | 2 (2-4) | 3 (3-3) | 3 (2-3) |
